# Development and testing the effectiveness and feasibility of a structured violence risk management intervention to support safety in psychiatric hospitals

**DOI:** 10.1192/j.eurpsy.2021.1081

**Published:** 2021-08-13

**Authors:** T. Lantta

**Affiliations:** Department Of Nursing Science, University of Turku, UNIVERSITY OF TURKU, Finland

**Keywords:** risk assessment, violence, Psychiatric hospital, user involvement

## Abstract

**Introduction:**

This presentation focuses on two major problems in psychiatric hospital care: patient violence and lack of patient engagement. Interventions already exist for managing patient violence. However, the challenge in using these interventions is poor integration to clinical practice and these methods do not entail elements of patient engagement.

**Objectives:**

The aim of the presentation is to give on overview of a project aiming to develop and test new structured intervention for violence risk management. Intervention aims to increase safety in care environments and engagement of patients.

**Methods:**

Intervention Mapping protocol together with staff and patients will be used in the project. Quasi-experimental design is used to test the intervention in 4 month period in two psychiatric hospital units.

**Results:**

By the end of the year 2020, development of the the new violence risk management intervention is nearly finished. The presentation will give an outline of the developed intervention and how staff and patient engagement in the development phases were ensured.

**Conclusions:**

The project described in this presentation is an example how a feasible violence risk management method can be developed together with staff and patients receiving psychiatric care. By ensuring engagement of the target groups, here staff and patients, it is possible to promote real integration of a new working method to psychiatric inpatient care. This project was funded by the Academy of Finland (316206).

